# Sequence-based Analysis of the *Vitis vinifera* L. cv Cabernet Sauvignon Grape Must Mycobiome in Three South African Vineyards Employing Distinct Agronomic Systems

**DOI:** 10.3389/fmicb.2015.01358

**Published:** 2015-11-30

**Authors:** Mathabatha E. Setati, Daniel Jacobson, Florian F. Bauer

**Affiliations:** Institute for Wine Biotechnology, Stellenbosch UniversityStellenbosch, South Africa

**Keywords:** wine yeasts, next-generation sequencing, grapevine mycobiome, microbial diversity, microbial terroir

## Abstract

Recent microbiomic research of agricultural habitats has highlighted tremendous microbial biodiversity associated with such ecosystems. Data generated in vineyards have furthermore highlighted significant regional differences in vineyard biodiversity, hinting at the possibility that such differences might be responsible for regional differences in wine style and character, a hypothesis referred to as “microbial *terroir.*” The current study further contributes to this body of work by comparing the mycobiome associated with South African (SA) Cabernet Sauvignon grapes in three neighboring vineyards that employ different agronomic approaches, and comparing the outcome with similar data sets from Californian vineyards. The aim of this study was to fully characterize the mycobiomes associated with the grapes from these vineyards. The data revealed approximately 10 times more fungal diversity than what is typically retrieved from culture-based studies. The Biodynamic vineyard was found to harbor a more diverse fungal community (*H* = 2.6) than the conventional (*H* = 2.1) and integrated (*H* = 1.8) vineyards. The data show that ascomycota are the most abundant phylum in the three vineyards, with *Aureobasidium pullulans* and its close relative *Kabatiella microsticta* being the most dominant fungi. This is the first report to reveal a high incidence of *K. microsticta* in the grape/wine ecosystem. Different common wine yeast species, such as *Metschnikowia pulcherrima* and *Starmerella bacillaris* dominated the mycobiome in the three vineyards. The data show that the filamentous fungi are the most abundant community in grape must although they are not regarded as relevant during wine fermentation. Comparison of metagenomic datasets from the three SA vineyards and previously published data from Californian vineyards revealed only 25% of the fungi in the SA dataset was also present in the Californian dataset, with greater variation evident amongst ubiquitous epiphytic fungi.

## Introduction

*Vitis vinifera* L. is an economically important crop plant that has been cultivated since ancient times. Throughout growth and development, the grapevines interact with a wide range of filamentous fungi and yeasts that colonize vegetative tissues and reproductive organs (Pancher et al., 2012). The fungal population comprises endophytic and epiphytic communities that may be pathogenic, neutral, or beneficial to the host (Pancher et al., 2012; Martins et al., 2014). Many studies employing culture-dependent and culture-independent approaches have shown that the grape berry endosphere is mainly colonized by ascomycetous filamentous fungi of the genera *Alternaria*, *Botryotinia*, *Epicoccum*, *Davidiella*, *Neofusicoccum*, and *Cladosporium* (Martini et al., 2009; Gonzalez and Tello, 2011). The endophytic fungi play a crucial role in plant health as they can retard the growth of detrimental phytopathogens (Martini et al., 2009). In contrast, the epiphytic fungal community comprises saprophytic filamentous fungi of the genera *Aspergillus*, *Penicillium*, *Rhizopus*, and obligate parasites including *Erysiphe necator* and *Plamospara viticola*, as well as oxidative and fermentative yeasts that influence wine fermentation processes and contribute to the aroma and flavor of wine (Diguta et al., 2011; Rousseaux et al., 2014). The yeast population on grape surfaces is mainly dominated by basidiomycetous yeasts of the genera *Cryptococcus*, *Rhodsporidium*, and *Rhodotorula* pre-véraison, while the ascomycetous yeasts, particularly species of the genera *Hanseniaspora*, *Metschnikowia*, and *Candida*, increase in numbers as the fruit ripens. The yeast-like fungus *Aureobasidium pullulans* is dominant throughout the berry development and has been shown to exist as both an endophyte and epiphyte (Martini et al., 2009). The presence of other yeast genera depends upon various factors including vineyard practices (Setati et al., 2012; Martins et al., 2014), disease pressure and the level of damage of the grapes (Barata et al., 2012).

Although many studies have been performed to describe both the endophytic and epiphytic fungal communities associated with grape berries, most are based on culture-dependent methods and either target the two groups separately, or are mainly focused on the yeast population and not the entire fungal population. Recently, metagenomic approaches have become an important tool for assessment of the grape microbiome. Bokulich et al. (2014) comprehensively examined the communities of both bacteria and fungi in crushed Chardonnay and Cabernet Sauvignon fruit in California using Illumina amplicon sequencing approaches and showed that the microbiomes not only differed by region, but were also conditioned by climate, year, and cultivar. Similarly, Taylor et al. (2014) demonstrated regional distinction in fungal communities in vineyards across New Zealand. The diversity of fungi associated with grapes and present in grape must were shown to resemble that present on leaves (Bokulich et al., 2014; Pinto et al., 2014), and the community composition is influenced by chemical treatments, agronomic practices, and climatic conditions (Bokulich et al., 2014; David et al., 2014; Pinto et al., 2014).

Metagenomic surveillances were shown to reveal greater diversity than other community fingerprinting methods and culture-based methods (David et al., 2014; Taylor et al., 2014). In fact, Taylor et al. (2014) suggested that culture-based methods might miss up to approximately 95% of the community in some samples. Consequently, these methods are increasingly becoming the preferred tool to evaluate the grape microbial community structures. The aim of the current study was therefore to employ a sequence-based metagenomic approach to better characterize fungal community structures associated with Cabernet Sauvignon grapes from three neighboring vineyards that employ different agronomic strategies and were shown through community fingerprinting and culture-based methods to harbor distinct communities. In addition, the fungal community structures associated with grape berries in South Africa and California (USA) were compared to determine continental distribution and prevalence of fungal species.

## Materials and Methods

### Grape Sampling and DNA Extraction

Cabernet Sauvignon grapes were collected from 3 vineyards located in the Polkadraai area of Stellenbosch, South Africa. The viticultural practices applied in these vineyards [referred to as biodynamic (BD), conventional (CONV) and integrated production of wine (IPW)], their lay-out and relevant characteristics are described in detail in Setati et al. (2012). The three vineyards are located next to each other; BD (33°57′39.33″ S 18°45′13.46″ E elev 183 m), CONV (33° 57′41.50″ S, 18°45′11.87″ E elev 179 m) and IPW (33°57′40.65″ S 18°45′08.23″ E elev 184 m). The CONV and BD vineyard had the same Cabernet Sauvignon rootstock (R101-14) while the integrated vineyard has rootstock R110-CS23A. Briefly, the BD vineyard applies sulfur, copper oxide as well as organic fungicide for control of powdery mildew and downy mildew while the integrated vineyard applies biofertilizers, mycorrhizae, as well as a combination of systemic and surface protectants for pest control. In contrast, the CONV vineyard mainly applies chemical fungicides and biofertilizers. The grapes were collected from the vineyards based on a sampling design described previously (Setati et al., 2012). From each vineyard 5 kg of grapes were collected from the selected sampling sites and pooled into a composite sample, hand de-stemmed and crushed under aseptic conditions in the laboratory. Only healthy undamaged grapes were used for the analysis. The chemical composition of the must was analyzed by Fourier Transform Infrared (FT-IR) spectroscopy using the GrapeScan 2000 instrument (FOSS Electric, Denmark). Fifty milliliters of grape must were collected immediately after crushing and used for DNA extraction. The grape must was centrifuged at 5000 rpm for 5 min and the pellet washed three times with a buffer comprising 0.15 M NaCl, 0.1 M EDTA, and 2% (w/v) Polyvinylpyrrolidone (Jara et al., 2008), followed by three washes with TE buffer (pH 7.6). DNA extraction was carried out according to Wilson (2003) with minor modifications. Briefly, the pellet was re-suspended in 2.3 ml TE buffer, followed by the addition of proteinase K, SDS, and 500 μl of fine glass beads. The mixture was vortexed for 3 min. A volume of 20 μl of a 10 mg/ml lysozyme solution was added and the mixture incubated at 37°C for 50 min. Then 400 μl of 5 M NaCl and 240 μl CTAB/NaCl (CTAB: Cetyl-methyl ammonium bromide) was added and the mixture was incubated for 10 min at 65°C, followed by phenol/chloroform/isoamyl extractions and precipitation with isopropanol.

### Sequencing Library Construction

Amplification of the ITS1-5.8S rDNA-ITS2 was performed using fusion primers consisting of the ITS1 (5′-TCCGTAGGTGAACCTGCGG-3′) and ITS4 (5′-TCCTCCGCTTATTGATATGC-3′) primers and Illumina MiSeq platform specific adaptor sequences. In a study comparing primers targeting the ITS1, ITS2, and whole ITS, Bokulich and Mills (2013) showed that no primer pair could accurately reconstruct the known taxonomic distribution of a mock community. Consequently, for the current study we chose to target the whole ITS region for better taxonomic assignment of reads. The PCR was performed in 25 μl reactions containing 1 × Ex-Taq buffer, 0.2 mM dTNPs, 0.25 μM of each primer and 100 ng DNA template. Triplicate reactions were performed for each DNA sample. Cycling conditions consisted of an initial denaturation at 94°C for 3 min, followed by 40 cycles of denaturation at 94°C for 30 s, annealing at 55°C for 30 s and extension at 72°C for 45 s; and a final extension of 10 min at 72°C. The PCR products were purified using the Zymoclean^TM^ Gel DNA recovery kit (The Epigenetics Company^TM^, Zymo Research, Inqaba Biotechnical Industries (Pty Ltd., South Africa) and quantified using the NanoDrop^TM^ 1000 spectrophotometer (Thermo Scientific). The amplicons from triplicate PCR reactions were combined at equal concentrations and used for Illumina library preparation and sequencing. Samples were subjected to standard quality control measures (fluorometric quantification and normalization). One nanogram of each amplicon pool was used in a standard indexing PCR protocol for a paired-end sequencing library (Nextera) and samples were sequenced using MiSeqV3 chemistry (2 × 300 reads).

### Data Analysis

Raw Illumina fastq files were uploaded onto the MG-RAST server (Meyer et al., 2008) and de-replicated (Gomez-Alvarez et al., 2009). The sequences were screened for plant (host-specific) DNA (Langmead et al., 2009) and low quality sequences with a Phred score below 30 were identified using the dynamic trimming (Cox et al., 2010) and removed. The Fastq join script was used to join overlapping paired-end reads. Since the ITS-5.8S region of some fungi is larger than 600 and would therefore not overlap, both joined reads and those that did not overlap were retained (i.e., no sequences were discarded) for further analysis. All sequences were processed for quality analysis. The resulting data sets were pre-screened using qiime-uclust (Edgar, 2010) clustered at 97% identity by picking the longest sequence within each cluster as a representative of that cluster. Taxonomic assignment was performed in MG-RAST using the Blast Like-Alignment Tool (BLAT) search against the M5RNA database with an *E*-value and similarity cut-off of 1e^-10^ and 99%, respectively, and a minimum alignment length of 150 bp. Pearson correlation was used to compare the taxa derived from forward reads (mainly representing partial ITS1-5.8S rDNA), reverse reads (mainly representing partial ITS2-5.8S rDNA) as well as the mix containing joined reads (representing both the partial and full ITS1-5.8S rDNA-ITS2). The MG-RAST accession codes for the libraries are: 4561567.3, 4561568.3, and 4561569.3. Classical ecology indices such as Shannon Wiener diversity index (*H′*) and Simpson dominance and diversity (*D*, *1-D*) were calculated using the free software package, PAST Version 3.0 (Hammer et al., 2001). The estimated richness was computed on a subsample of 20000 reads. Following taxonomic assignment the data was transformed into a presence/absence matrix and analysis of variance (ANOVA) was performed. A Perl program was written to create a weighted co-occurrence network depicting the species present in and across vineyards. The resulting network was visualized with Cytoscape (Shannon et al., 2003). In addition, the data generated in the current study was compared to yeast isolates that we obtained in a parallel study from the three grape musts by culture-based methods (Bagheri et al., 2015) and also to the metagenomic data generated from grape musts obtained from vineyards in different regions of California (Bokulich et al., 2014). Composite lists of the of the fungal species in the SA and California amplicon sequencing data were compiled and compared with the yeast isolates using Venn’s diagrams, constructed on http://bioinformatics.psb.ugent.be/webtools/Venn/.

## Results

### Sequence Analysis and Taxonomic Assignment

**FIGURE 1:**
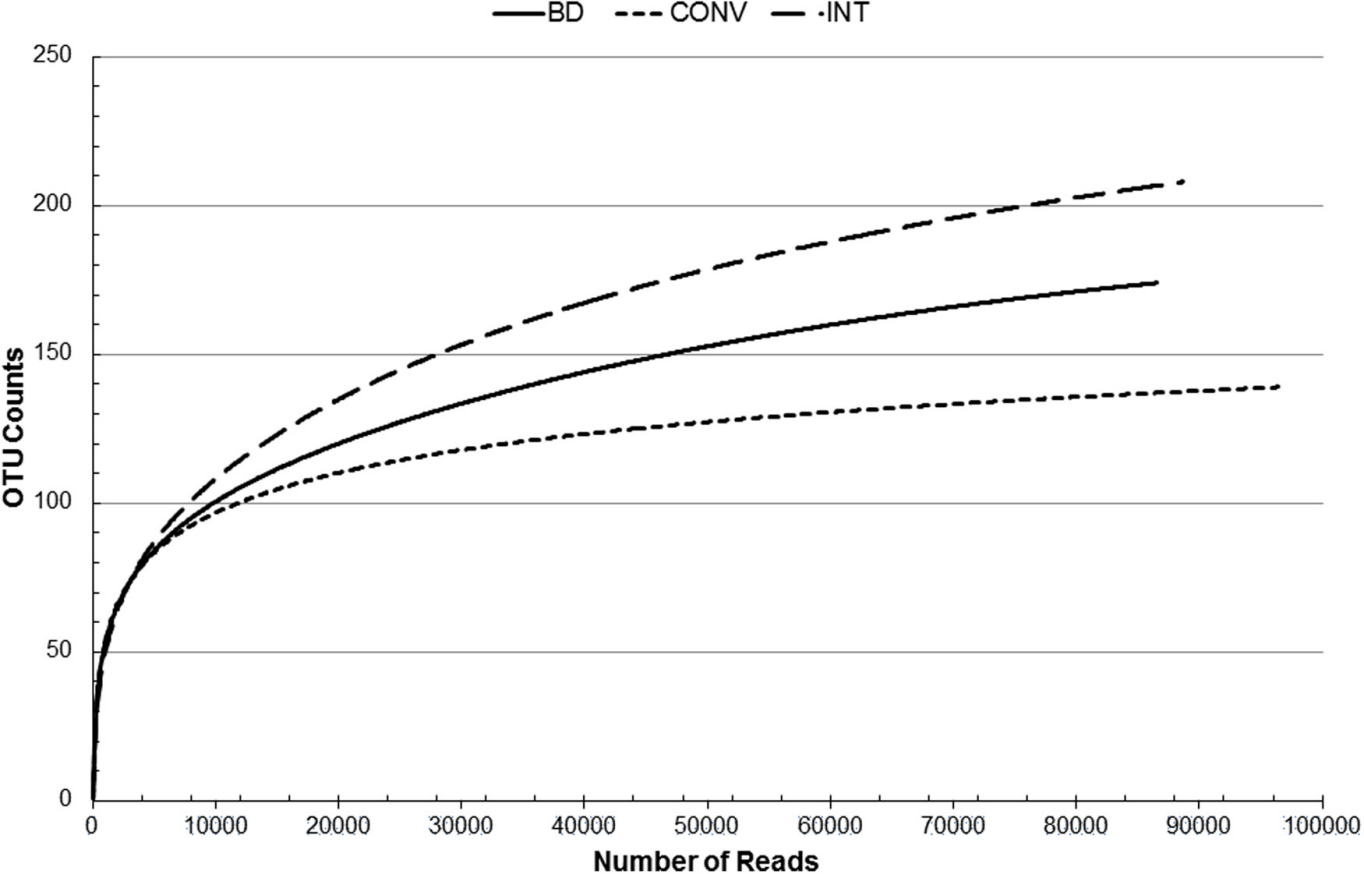
**Rarefaction analysis of community richness estimates based on sequences that passed Phred quality score of 30**.

We previously assessed the grape berry associated diversity in the three vineyards and demonstrated using Automated Ribosomal Intergenic Spacer analysis (ARISA) that the fungal community structure was distinct (Setati et al., 2012). In addition, data derived from culture dependent microbiological analysis suggested that the BD vineyard had a more diverse fungal community than the CONV and integrated (IPW) vineyard (Setati et al., 2012). In the current study, Illumina paired end sequencing was used to explore the fungal biota (mycobiome) of the different vineyard samples. ITS1-5.8S rDNA-ITS2 libraries were generated from genomic DNA extracted from freshly crushed grape must samples prepared from composite samples. Chemical analysis of the musts shows differences in the ripeness level of the grapes (**Supplementary Table S1**). For the sequence data, quality filtering removed 29% of the reads from the BD and CONV libraries while only 24% was removed from the IPW library (**Supplementary Table S2**). The Streptophyta (data not included in further analysis) only accounted for less than 1% of the total sequence data in the three libraries. Unassigned sequences accounted for 295, 777, and 153 reads of the total reads in the BD, CONV, and IPW libraries, respectively. Our data revealed good correlation between taxonomic assignments from the forward reads (mainly containing partial ITS1-5.8S sequences) and the data sets containing all reads (i.e., joined ITS1-5.8S-ITS2) and single reads (partial ITS1-5.8S and ITS2-5.8S), while the reverse reads (containing partial ITS2-5.8S sequences) from the BD and IPW showed poor correlation with the forward and joined reads (**Supplementary Table S3**). Based on this, we chose to use the dataset containing both joined and single reads. Therefore, for yeast species with short ITS-5.8S rRNA regions the taxonomic assignment was based on the full ITS1-5.8S rRNA-ITS2 gene while for other yeasts only the partial gene would have been used. Rarefaction curves showed that the sampling depth and sequencing coverage were good for all three samples, especially for the CONV sample which had clearly reached a plateau (**Figure 1**). Diversity analysis revealed that the BD library comprised a more diverse mycobiome with low dominance (*H′* = 2.6; *D* = 0.11) followed by the CONV (*H′* = 2.1; *D* = 0.21), while the IPW had the lowest diversity and highest dominance (*H′* = 1.77; *D* = 0.3). ANOVA analysis performed on the presence/absence transformed data showed that the community in the three vineyards was significantly different (*p* = 0.025).

**FIGURE 2:**
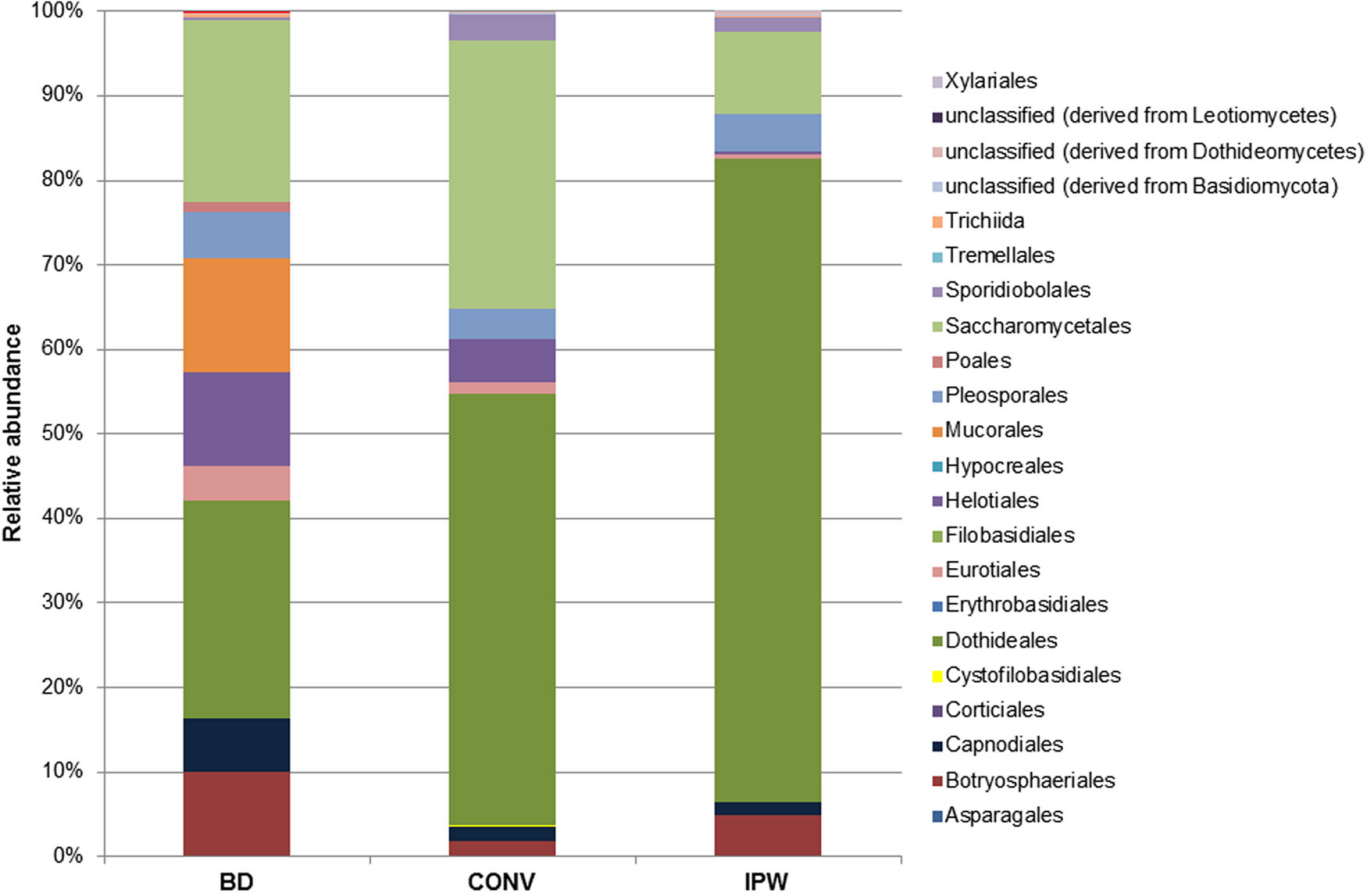
**Distribution of fungal species recovered across the orders of Ascomycetes, Basidiomycetes, and Zygomycetes**.

**FIGURE 3:**
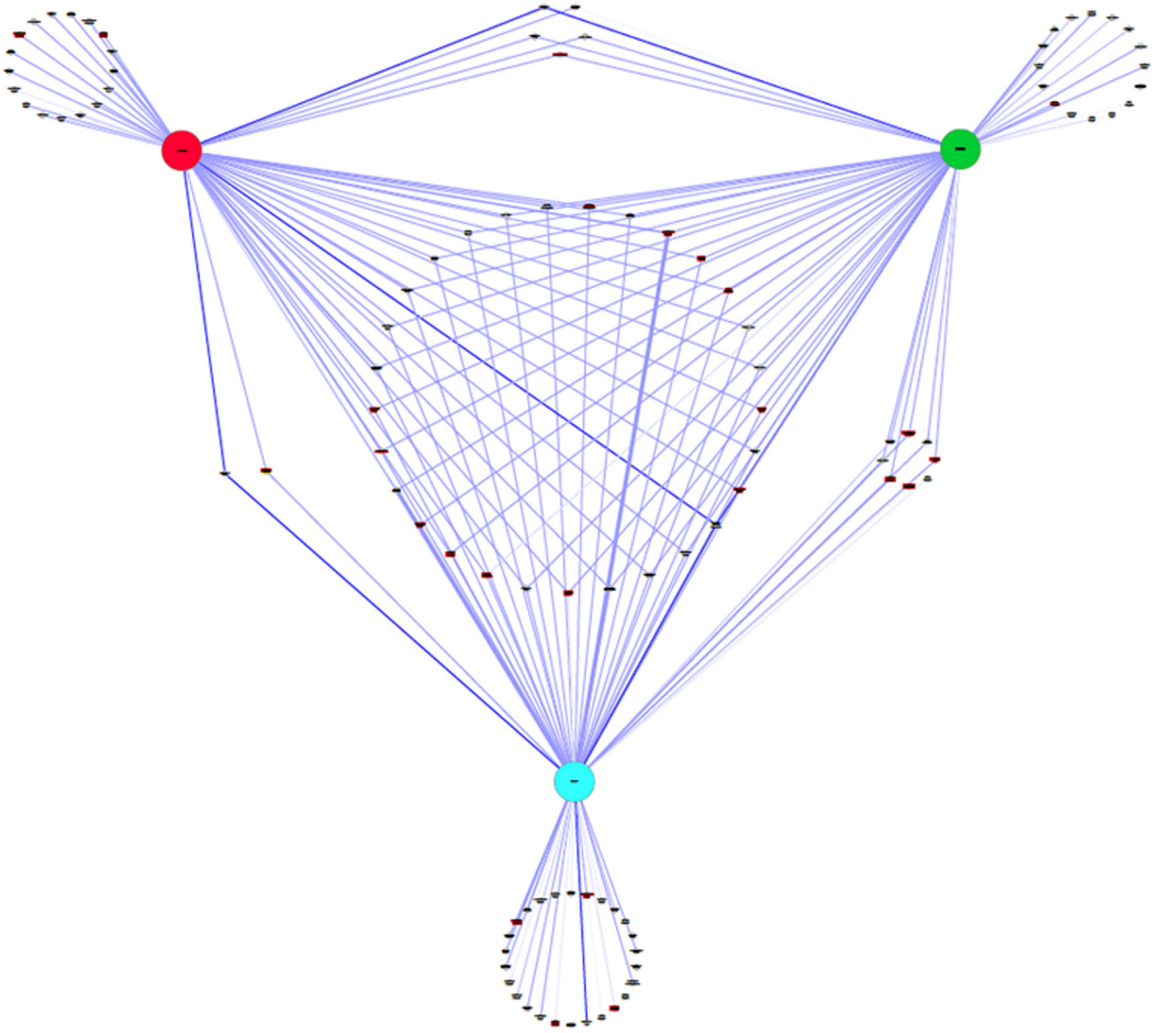
**A weighted co-occurrence network of the fungal communities in the grape must prepared from grapes obtained from the biodynamic (BD; green), conventional (CONV; red) and integrated (blue) vineyard**.

Taxonomic assignment was performed using the MG-RAST pipeline. The data indicated some overall similarities in the species composition, but also significant differences. The Ascomycota was found to be the predominant phylum represented in all three grape mycobiomes, but their total contribution varied significantly between 79 and 98% of the total fungal population. In contrast, the Basidiomycota which is commonly the dominant phylum on unripe berries only accounted for 0.4% of the population in the BD vineyard, while in the CONV and IPW vineyard it represented 3.4 and 2%, respectively. In contrast, the BD grape must displayed a high incidence of fungi from the phylum Zygomycota (20%) while in the CONV and IPW vineyard this phylum represented less than 0.1% of the fungal population. Further analysis shows that fungi of the order Dothidiales were dominant across the three libraries. The Saccharomycetales were also present in high levels in the BD and CONV libraries, while the Botryosphaeriales were the second most dominant in the IPW library (**Figure 2**). In addition, in the BD must sample the Mucorales were present at the same level as the Saccharomycetales accounting for 20% of the taxa. Dominant ascomycetous filamentous fungi included members of the genera *Alternaria*, *Botryotinia*, *Cladosporium*, *Davidiella*, *Kabatiella*, *Neofussicoccum*, *Pleospora*, and the yeast-like fungus *A. pullulans*, while *Rhodosporidium* sp., *Sporobolomyces* sp. and *Rhodotorula* sp. where the predominant basidiomycetous fungi. Twenty nine fungal species were common across the three vineyards (**Figure 3**). There were evidently more species shared between the BD and IPW vineyard, than between the BD and CONV, or CONV and IPW.

### Distribution of the Filamentous Fungal Taxa

**FIGURE 4:**
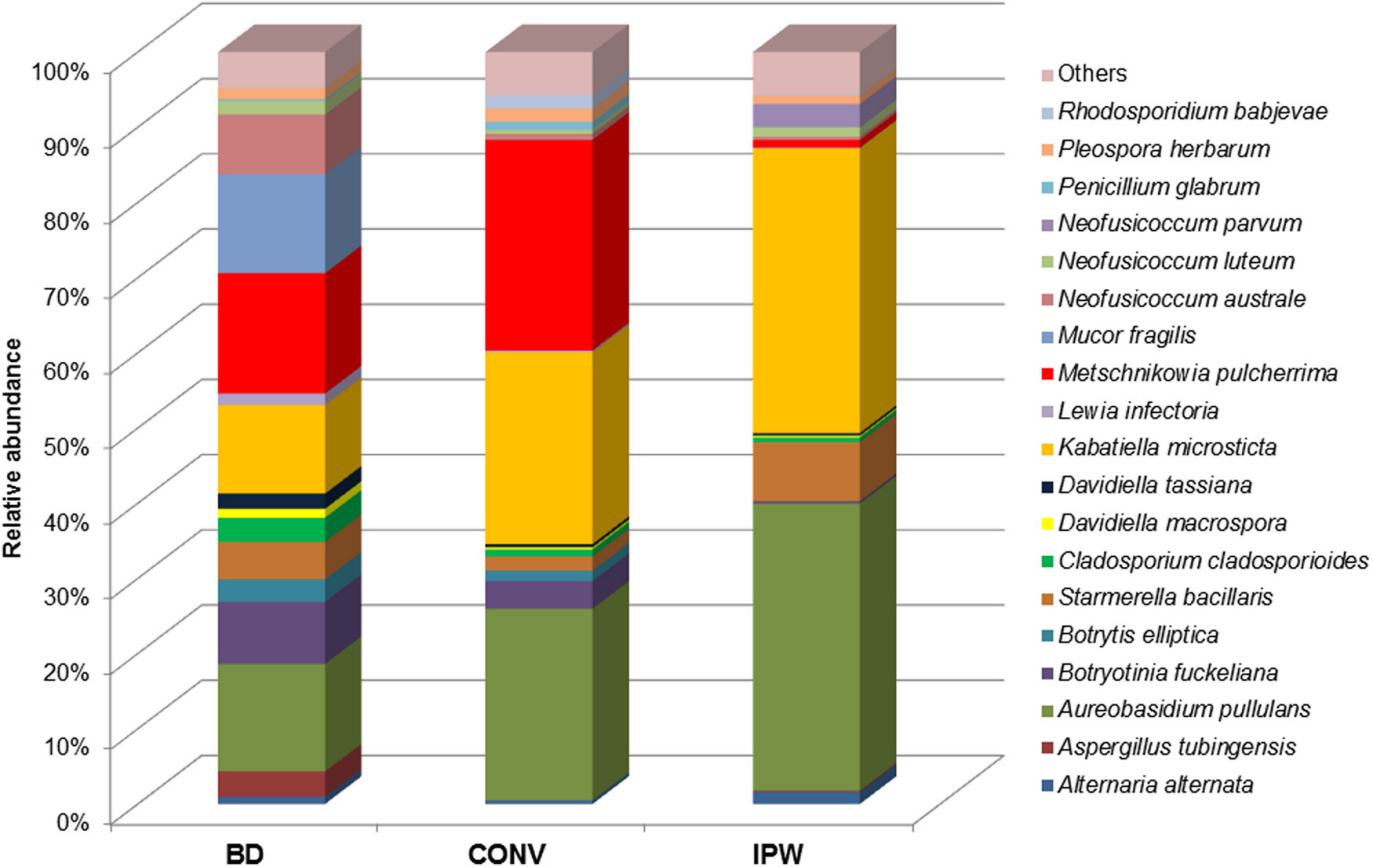
**The frequency of occurrence of the abundant fungal taxa as well as major grapevine associated taxa**.

Our data revealed two fungi as the most abundant taxa in the must samples from the three vineyards. The yeast-like fungus *A. pullulans*, which has been reported as both an endophyte and an epiphyte of grapevine, accounted for 13, 25, and 38% of the total population in the BD, CONV, and IPW vineyard, respectively. Similarly, *Kabatiella microsticta* which is closely related to *A. pullulans*, accounted for 11, 25, and 38% of the population in the BD, CONV, and IPW vineyard must sample, respectively. Amongst common grapevine endophytes, *Botryotinia fuckeliana*, *Neofusicoccum australe*, *Cladosporium cladosporioides*, *Davidiella tassiana*, *Lewia infectoria*, and *Mucor* sp., were abundant in the BD vineyard must, while the IPW must displayed a more diverse *Neofusicoccum* community, with *N. parvum* being the dominant species of this genus. *Phoma herbarum* and *Diplodia seriata* were more dominant in the CONV vineyard (**Figure 4**). Fungi that were abundant amongst typical epiphytic taxa in the three vineyards were *Penicillium brevicompactum*, *P. corylophilum*, *P. glabrum* and *Pleospora herbarum*. In contrast, *Aspergillus tubingensis* was only present in BD and IPW, while *Botrytis elliptica* was present only in BD and CONV. The CONV exhibited a lower diversity of grapevine phytopathogens compared to the BD and IPW. Some of the fungi detected in the mycobiome were not previously known to associate with grapevine such as *Ascochyta rabiei*, *Aschochyta fabae*, *P. sojicola* (synonym, *A. sojicola*), *Lophodermium pinastri*, and *Sphaeropsis sapinea* (synonym, *D. pinea*). These fungi were, however, present at levels below 1%. Overall, fungi that are potential grapevine pathogens accounted for 50% of the total population in the must from the BD vineyard, while in the CONV and IPW, they accounted for 10 and 8%, respectively.

### Analysis of the Yeast Community

**FIGURE 5:**
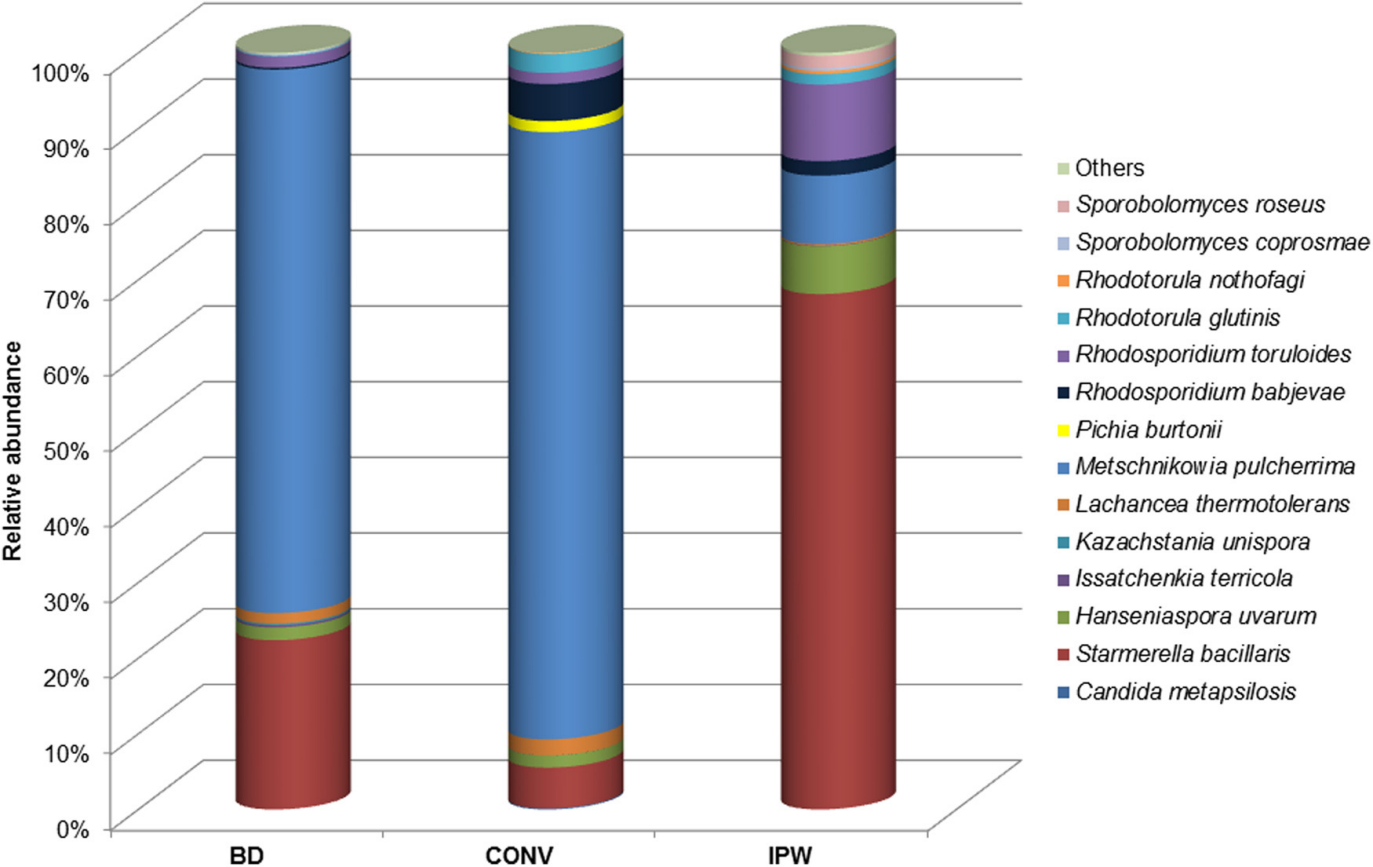
**Relative abundance of yeast species frequently encountered in the wine microbial consortium**.

**FIGURE 6:**
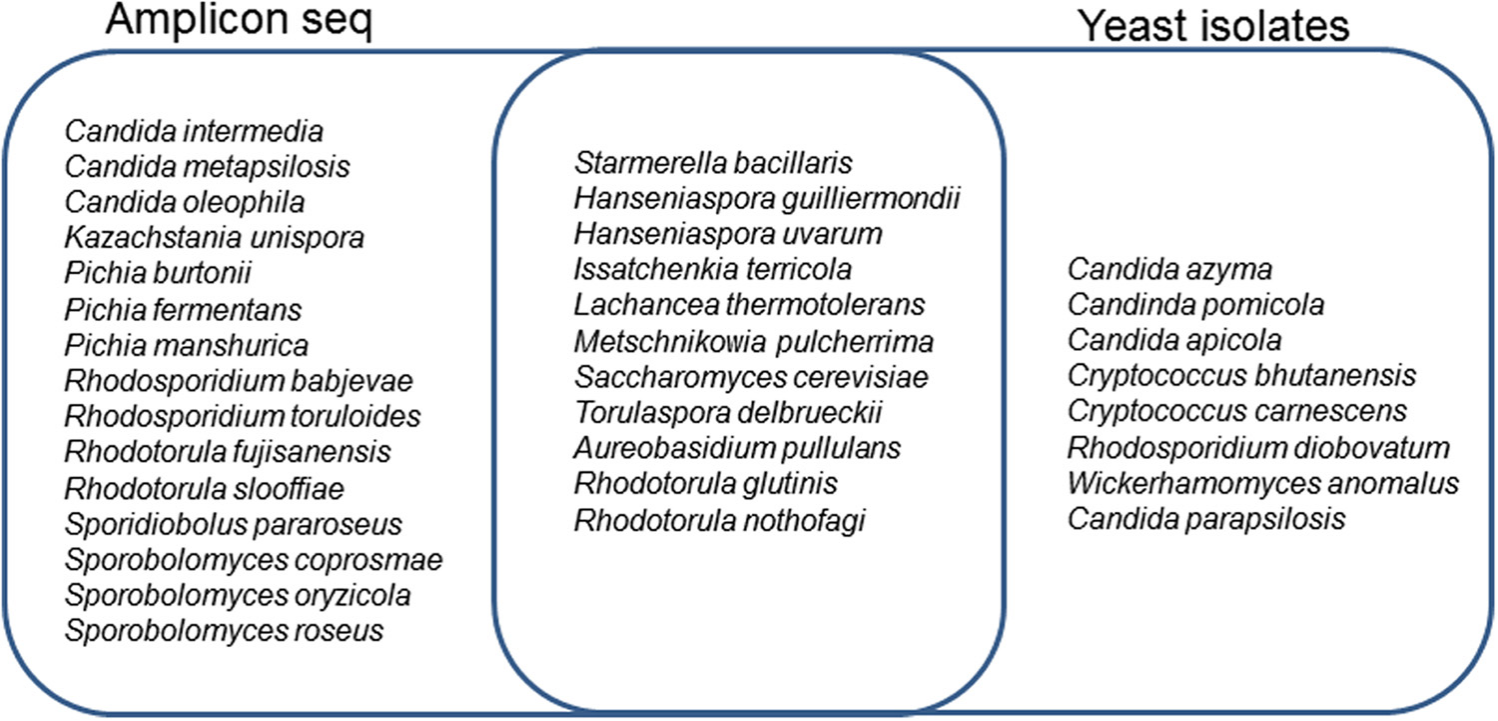
**A Venn diagram showing yeast species distribution between the amplicon sequencing data and cultivated yeast isolates from the BD, CONV, and IPW vineyard must samples**.

Yeasts that constitute the wine microbial consortium have been grouped into previously described categories: (i) oligotrophic oxidative yeasts, e.g., (*Cryptococcus* sp., *A. pullulans*, *Rhodosporidium* sp., *Sprobolomyces* sp.), (ii) copiotrophic oxidative and weakly fermentative yeasts, e.g., (*Candida* sp., *Pichia* sp., *Hanseniaspora* sp., *Metschnikowia pulcherrima*, *Rhodotorula glutinis*, *Lachancea thermotolerans*), and (iii) copiotrophic strongly fermentative yeasts, e.g., (*Torulaspora delbrueckii*, *Saccharomyces* sp., *Zygosaccharomyces* sp.), (Ocón et al., 2010; Barata et al., 2012). These groups of yeasts accounted for 22, 35, and 12% of the total fungal diversity in the BD, CONV, and IPW, grape must samples, respectively. The oxidative yeasts mainly comprised *Sporobolomyces* sp., *Rhodosporidium* sp., and *Rhodotorula* sp., which were only present at low levels (**Figure 5**). *M. pulcherrima* was the most dominant weakly fermentative yeast in the BD and CONV mycobiome, while *Starmerella bacillaris* (synonym, *Candida zemplinina*) was the most dominant in the IPW mycobiome. *Hanseniaspora uvarum* was present in similar amounts in the three mycobiomes. The strongly fermentative yeasts were generally present at very low levels. Amongst them, *L. thermotolerans* was detected in higher levels in the BD and CONV mycobiome, *T. delbrueckii* was only detected in the IPW mycobiome while *Kazachstania unispora* was only detected in the BD mycobiome and *Saccharomyces cerevisiae* only in the CONV mycobiome (**Figure 5**). Overall, 11 fermentative yeast species were detected in the BD mycobiome while 8 were detected in the CONV and 9 in the IPW mycobiomes. A comparison of the sequence data with the yeasts isolated from the same must samples shows the most commonly isolated yeasts could be detected by both methods with 11 species shared between them (**Figure 6**).

### Comparative Analysis of SA and California Data

**FIGURE 7:**
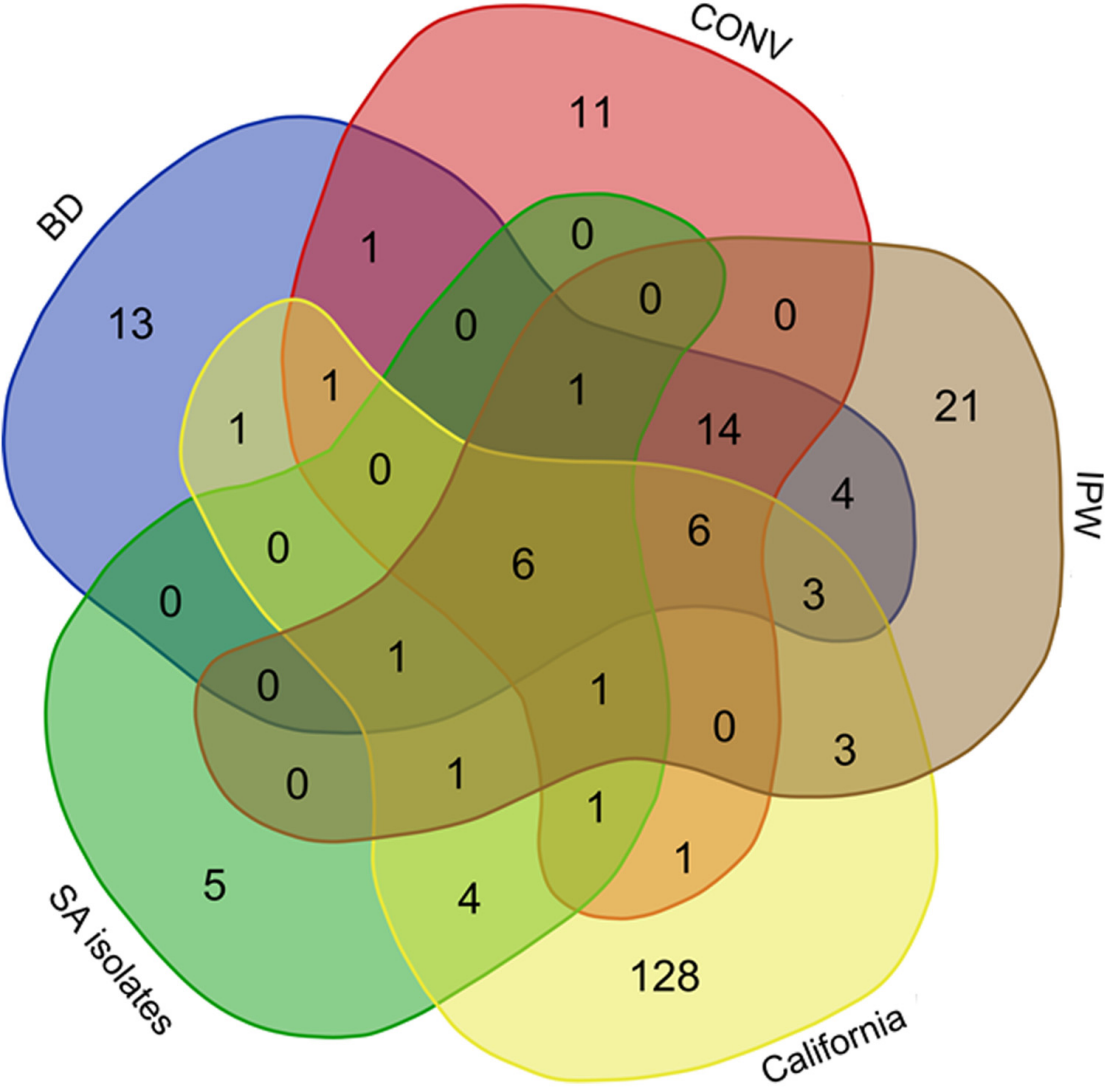
**A Venn diagram representing comparison of yeasts isolated from the BD, CONV and IPW vineyard must samples in SA against the species obtained from amplicon sequencing data derived from the SA and Californian grape must samples**.

Composite lists of the fungal species detected in the grape musts from the three SA vineyards and those found in Californian vineyards through Illumina amplicon sequencing were generated and matched against the list of yeast isolates from the SA vineyards. The data revealed vast differences in fungal diversity detected through amplicon sequencing from the two countries with only 29 fungal species shared between the two data sets (**Figure 7**). Fifteen species were common between the SA and California mycobiomes, while 10 species were common across SA yeast isolates, SA mycobiomes and California mycobiomes. An additional four species were common between the SA isolates and California mycobiomes. The common fungi can be broadly grouped into (i) yeasts typically found in the wine microbial consortium such as *L. thermotolerans*, *T. delbrueckii*, *S. bacillaris*, *S. cerevisiae*, *I ssatchenkia terricola*, *H. uvarum*, and *Hanseniaspora guilliermondii*, (ii) genera that are frequent components of plant endophyte surveys such as *Alternaria*, *Davidiella*, *Lewia*, *Phoma*, *Aureobasidium*, and *Epicoccum* and (iii) ubiquitous epiphytes such as *Penicillium* and *Aspergillus* species. Our data also revealed one yeast species isolate (*M. pulcherrima*) that was detected only in the musts from SA mycobiomes and not the California mycobiomes (**Figure 7**).

## Discussion

The diversity of yeast and fungi associated with the grape berry and grape must have been the focus of many studies in the past. However, most of these studies have mainly relied on culture-dependent methods to define the diversity. Recently, culture-independent methods including ARISA, DGGE, and CE-SSCP have been employed especially in comparative studies as they provide a better overview of microbial community structures in different samples. However, confident identification of taxa represented in the community fingerprints is not always easy or reliable. Consequently, metagenomic approaches are the methods of choice for unraveling the microbiome associated with different ecosystems. In the current study, Illumina sequencing of the ITS1-5.8S rDNA-ITS2 gene sequences directly amplified from grape must samples derived from a CONV, integrated and BD vineyard. Our data show that the Ascomycota are the most dominant phylum constituting the grape must mycobiome. This is in agreement with data reported by Bokulich et al. (2014) and also with cultivation based studies that have shown that the grapevine fungal endophytes mainly comprise ascomycetous fungi while the epiphytic community has also been shown to shift from a basidiomycetes dominated community at berry-set to an ascomycetes dominated community at full ripeness (Prakitchaiwattana et al., 2004; Renouf et al., 2005, 2007). The BD vineyard displayed a higher incidence of Zygomycetes mainly represented by *Mucor* and *Rhizopus* species. Pinto et al. (2014) recently reported such fungi belonging to early diverging fungi to account for close to 28% of the total mycobiota of grapevine leaves. *Mucor* sp. are most known to cause post-harvest rot in table grapes but rarely in wine grapes (Kassemeyer and Berkelmann-Lohnertz, 2009). Our previous data using ARISA analysis demonstrated that the epiphytic fungal community associated with the three vineyards was distinct from each other (Setati et al., 2012).

The current data revealed that members of the order Dothidiales were the most abundant in the three vineyards, albeit with very significant differences, since the two most prominent species, *A. pullulans* and *K. microsticta* accounted for 24, 50, and 76% in the BD, CONV, and IPW vineyard, respectively. *A. pullulans* is a common inhabitant of the grapevine ecosystem and has previously been shown to be present amongst both the endophytic and epiphytic fungal communities. In our previous study, we also found this yeast-like fungus to be the most abundant yeast isolated from the grape surface where it accounted for more than 50% of the yeast isolates (Setati et al., 2012). *K. microsticta* on the other hand, has never been isolated from grape vine before. In fact, members of this genus have not been successfully cultured and are only known from their sporodochial stages (Zalar et al., 2008). Importantly, the genus *Kabatiella* is a plant pathogen known to cause leaf spot on specific plant species. Its presence in grape must might be due to the transfer of spores from the epiphytic microbiota of the leaves to the grape berries. This fungus has not been shown to be a pathogen of grapevine. It would therefore be highly relevant to investigate whether this pathogen can impact on grapevine, and whether its presence is of wider relevance for the SA and global wine industry, or whether its occurrence is locally restricted. Interestingly, other *Kabatiella* sp. have been found associated with the Proteaceae family, characteristic of the fynbos biome endemic to the Western Cape province of South Africa (Taylor and Crous, 2000), suggesting that they might indeed be common members of the regional plant microbiota.

The BD grape must exhibited a higher incidence of phytopathogenic molds with potential to cause post-harvest rot. Some of these fungi, e.g., *Alternaria* sp. and *Cladosporium* sp., have previously been isolated from the grape endosphere (Pancher et al., 2012). Importantly, only healthy grapes have been used for our analysis, and the overall health status of all three vineyards at harvest appeared visually similar (i.e., no apparent diseased state). This not suggests that these fungi find it easier to colonize vineyards that are not treated with fungicides, but that their increased presence in the vineyard may itself not be problematic. Indeed, Dugan et al. (2002) demonstrated that grape berries were progressively infected with quiescent fungi, mainly members of the genera *Alternaria*, *Aureobasidium*, *Cladosporium*, and *Ulocladium*. Invasion by the fungus may occur via the stigma and style, resulting in latent infection of the berry. By harvest time, as much as 25–78% of the grape clusters may be colonized with various fungi including *B. cinerea/B. fuckeliana* (Dugan et al., 2002). These fungi, therefore may reside in the berry without causing any disease. The overall higher biodiversity within the BD vineyard may indeed act as a protective element.

Only the musts from the IPW vineyard contained a diverse group of *Neofussicoccum* species. These fungi, especially *N. parvum* are opportunistic pathogens of grapevine, proven to cause *Botryosphaeria* dieback. *N. parvum* which was only detected in the IPW vineyard must and *N. australe* which was most dominant in the BD vineyard must, are some of the most virulent species in South Africa (van Niekerk et al., 2004). Another Botryosphaeriaceous fungus detected in the IPW mycobiome was *Lasiodiplodia theobromae*, which has been reported as the most virulent of this group of fungi (Úrbez-Torres and Gubler, 2009). Although these fungi are often isolated from the woody grapevine, they have also been isolated from grapes (Yan et al., 2013). *L. theobromae* typically infects grape berries during véraison. However, the germination of Botryosphaeriacious fungi is highly dependent on temperature and humidity (Yan et al., 2013). Interestingly, *Ampelomyces quisqualis* was detected in the mycobiome of the IPW vineyard. This fungus is a naturally occurring mycoparasite of several powdery mildew species and is used as a biocontrol agent against *E. necator* and other powdery mildew species (Falk et al., 1995; Angeli et al., 2009). Some of the fungi detected in the mycobiome of the different vineyards are known as pathogens of other plants. These include *A. fabae* and *A. rabiei* which are known to cause blight disease in chick pea, wheat, barley, oats, rye, triticale, and turf grasses and may have been transferred from neighboring plants or plants such as oats which are commonly used as cover crops in the vineyard.

Common wine yeasts were found in the mycobiome from the three vineyards. *M. pulcherrima* and *S. bacillaris* were the most abundant weakly fermentative yeasts, followed by *H. uvarum*. Most of the other fermentative yeasts could be detected albeit at low levels. These included various *Candida* sp., *Pichia* sp., *L. thermotolerans*, *T. delbrueckii*, and *S. cerevisiae*. *K. unispora* was only detected in the must from the BD vineyard. Members of this genus were previously isolated amongst the epiphytic community in the same vineyard (Setati et al., 2012). Our data show that *S. bacillaris* is most abundant in the must with the highest sugar level, which is consistent with previous studies that have shown this yeast to be dominant in high sugar musts (Tofalo et al., 2009). This suggests that some of the differences in the three must samples can in part be attributed to differences in ripeness levels. Fermentations performed on the must from the three vineyards showed that the non-*Saccharomyces* yeast species that were already well represented in the must, persisted longer in fermentation (Bagheri et al., 2015). However, our data show disparity between culture-based method and high throughput amplicon sequencing with regard to the yeasts retrieved. For instance, different basidiomycetous yeasts of the genera *Rhodosporidium* and *Rhodotorula* were detected using culture-based method compared to those detected in the metagenome. For instance, in the metagenomic datasets *Rhodosporidium babjevae* and *R. toruloides* were detected, while the culture-based approach found *R. diobovatum*. In addition, yeasts such as *C. parapsilosis* and *Wickerhamomyces anomalus* previously shown to dominate fermentations in the BD and IPW must, respectively (Bagheri et al., 2015), could not be detected in the metagenomic data even though they were found to account for at least 10% of the initial population in the must. Similar disparities between culture-based methods and direct sequencing were reported by David et al. (2014) where for instance, *Sporobolomyces roseus* and *Bulleromyces albus* were found to account for 18–21% of the population in the middle of fermentation by culture-based method but could not be detected in the sequence data, while *T. delbrueckii* was found to be 15.9% of the population in one fermentation through direct sequencing but was not retrieved by culture-based methods. The reasons for such disparities could differ from species to species and might include DNA extraction biases in complex communities, PCR amplification bias, better cultivability as well as rapid growth for some species. The ratio of weakly fermentative to strongly fermentative yeasts was shown to influence fermentation rate. Pinto et al. (2014) demonstrated that some of the fermentative species of the genera *Saccharomyces*, *Hanseniaspora*, and *Metschnikowia* were present in the microbiome of leaves proving these organisms to be natural colonizers of the vine even before the appearance of the grape berries.

The data generated in the current study revealed huge differences in fungal assemblages between SA and Californian vineyards with approximately 25% of fungal species present in SA mycobiomes detected in the Californian mycobiomes. This was surprising especially since the California dataset covers an extensive number of vineyards which potentially should increase the probability of finding similar species when matching the SA dataset to the California data. However, given this difference in the community composition is probably acceptable given that these are cross-continental comparisons. The common fungal species mostly represented plant endophytes with antifungal properties useful against several plant diseases, as well as common constituents of the wine microbial consortium that drive fermentation processes. This suggests that there are only minor variations in resident mutualistic endophytes of grapevine, and the existence of a core group of species defining vineyard microbial ecosystem. The data show that there are two groups of fungal endophytes associated with *V. vinifera*. The first group comprises *Alternaria tenuissima*, *D. tassiana*, *Epicoccum nigrum*, *L. infectoria*, *Massarina corticola*, *P. herbarum*, and *Stemphylium* sp., which are intimately associated with *V. vinifera* globally, while the second group is characterized by fungi that are “host neutral” (i.e., generalist fungal pathogens) e.g., *D. seriata*, *N. parvum*, *N. australe*, and *L. theobromae*, that maybe horizontally transmitted between plant species and whose host affinity is strongly influenced by the environment (Slippers and Wingfield, 2007). This second group largely comprises botryosphaeriaceous fungi that have an endophytic phase and a pathogenic phase which can lead to rapid development of disease following the onset of stress due to factors such as extreme weather conditions (Slippers and Wingfield, 2007). Surprisingly, 9 species of this group of fungi were detected in SA vineyards while only 1 was detected in the Californian data sets. In contrast, a higher incidence of leaf spot inducing saprophytic fungi of the genera *Leptosphaeria*, *Phaeosphaeria*, and *Leptosphaerulina* was apparent in Californian vineyards. These findings highlight critical differences in plant pathogenic fungal clusters. Regarding yeasts of oenological relevance, similar yeast species could be detected across SA and California samples. Most of the species were also retrievable by cultivation, suggesting that strain variation as well as the combination and concentrations of individual species and strains are pivotal in determining stylistic distinction.

Overall, the current study shows the highly significant differences (*p* = 0.025) in fungal species assemblages between neighboring vineyards. Also, the data reveal interesting groupings of fungi and major distinctions in the *V. vinifera* mycobiomes across continents but also delineates a group of species that could be host specific endophytes. However, more data will be necessary to confirm such information. Most importantly, our data highlight critical differences in plant pathogenic fungal clusters. An in-depth investigation into these clusters could make significant contributions toward developing targeted control strategies that are focused on managing the most prevalent phytopathogens in a given region. Considering that the Californian study extended over large regions, it might appear surprising that the species overlap with our data is relatively small. The data therefore suggest that fungal ecosystem diverge very significantly according to region and vineyard, further supporting the idea of a microbial *terroir* of relevance to wine style and wine quality. Impact of farming practices also appears highly relevant in shaping fungal biodiversity, in particular when considering that the composite samples were representative of entire neighboring vineyards of the same age sampled at the same time.

## Conflict of Interest Statement

The authors declare that the research was conducted in the absence of any commercial or financial relationships that could be construed as a potential conflict of interest. The reviewer Carmen Portillo and handling Editor Alberto Mas declared their shared affiliation, and the handling Editor states that the process nevertheless met the standards of a fair and objective review.
