# Supplementary material for: Sequence-based Analysis of the Vitis vinifera L. cv Cabernet Sauvignon Grape Must Mycobiome in Three South African Vineyards Employing Distinct Agronomic Systems
Source: Front Microbiol. 2015 Nov 30;6:1358. doi: 10.3389/fmicb.2015.01358 (PMC4663253; doi:10.3389/fmicb.2015.01358)
Supplement: Supplementary file 1 [file Table_1.DOCX]

**Table S1:** Chemical composition of the grape must samples

| **Parameter** | **Biodynamic** | **Conventional** | **Integrated** |
| --- | --- | --- | --- |
| **Glucose/Fructose (g/L)** | 239 ± 0.20 | 210 ± 0.40 | 249 ± 0.30 |
| **°Brix** | 23.4 ± 0.00 | 20.7 ± 0.00 | 24.3 ± 0.02 |
| **Titratable acidity (g/L)** | 3.23 ± 0.01 | 3.85 ± 0.02 | 2.37 ± 0.04 |
| **pH** | 3.61 ± 0.001 | 3.35 ± 0.02 | 3.66 ± 0.002 |
| **Tartaric acid (g/L)** | 3.10 ± 0.05 | 2.30 ± 0.09 | 3.20 ± 0.03 |
| **Malic acid (g/L)** | 0.90 ± 0.005 | 1.40 ± 0.10 | nd |
| **Volatile acidity (g/L)** | 0.4 ± 0.001 | 0.31 ± 0.02 | 0.32 ± 0.01 |

nd: not detected
