# Supplementary material for: Sequence-based Analysis of the Vitis vinifera L. cv Cabernet Sauvignon Grape Must Mycobiome in Three South African Vineyards Employing Distinct Agronomic Systems
Source: Front Microbiol. 2015 Nov 30;6:1358. doi: 10.3389/fmicb.2015.01358 (PMC4663253; doi:10.3389/fmicb.2015.01358)
Supplement: Supplementary file 2 [file Table_2.DOCX]

**Table S2**. Statistical analysis of the sequence reads obtained from three metagenomic data sets derived from the Biodynamic (BD), Conventional (Conv) and Integrated (IPW) vineyard must samples prepared from Cabernet Sauvignon grapes.

| Statistical parameter | BD library | Conv library | INT library |
| --- | --- | --- | --- |
| Number of reads | 86,513 | 96,329 | 88,685 |
| Total number of bases (bp) | 29,087,037 | 32,900,613 | 31,604,360 |
| Mean read length (bp) | 336 ± 80 bp | 341 ± 83 | 356 ± 110 |
| Number of reads post QC | 61,273 | 68,385 | 67,101 |
| Total number of bases post QC (bp) | 10,751,714 | 13,433,357 | 12,124,193 |
| Mean read length post QC (bp) | 175 ±102 bp | 196 ± 108 | 180 ± 101 |
| % G + C | 45 ± 7% | 46 ± 5% | 49 ± 5 |
|  |  |  |  |
