# Supplementary material for: Sequence-based Analysis of the Vitis vinifera L. cv Cabernet Sauvignon Grape Must Mycobiome in Three South African Vineyards Employing Distinct Agronomic Systems
Source: Front Microbiol. 2015 Nov 30;6:1358. doi: 10.3389/fmicb.2015.01358 (PMC4663253; doi:10.3389/fmicb.2015.01358)
Supplement: Supplementary file 3 [file Table_3.DOCX]

**Table S3.** Pearson correlation matrices comparing taxonomic assignment using forward (ITS1-5.8S), reverse (ITS2-5.8S) or all reads including forward, reverse and joined paired reads (ITS1-5.8S-ITS2)

| **Biodynamic** | | | |
| --- | --- | --- | --- |
| Variables | BD Forward | BD Reverse | BD-All reads |
| BD Forward | 1 | 0.553 | 0.915 |
| BD Reverse | 0.553 | 1 | 0.612 |
| BD-All reads | 0.915 | 0.612 | 1 |
| **Conventional** | | | |
| Variables | CONV Forward | CONV Reverse | CONV-All reads |
| CONV Forward | 1 | 0.622 | 0.975 |
| CONV Reverse | 0.622 | 1 | 0.773 |
| CONV-All reads | 0.975 | 0.773 | 1 |
| **Integrated** | | | |
| Variables | IPW Forward | IPW Reverse | IPW-All reads |
| IPW Forward | 1 | 0.985 | 0.992 |
| IPW Reverse | 0.985 | 1 | 0.997 |
| IPW-All reads | 0.992 | 0.997 | 1 |
